# Diet quality and physical or comprehensive frailty among older adults

**DOI:** 10.1007/s00394-022-02819-w

**Published:** 2022-02-13

**Authors:** Daiki Watanabe, Kayo Kurotani, Tsukasa Yoshida, Hinako Nanri, Yuya Watanabe, Heiwa Date, Aya Itoi, Chiho Goto, Kazuko Ishikawa-Takata, Misaka Kimura, Motohiko Miyachi, Yosuke Yamada, Kyoto-Kameoka Study Group

**Affiliations:** 1 Department of Physical Activity Research, National Institute of Health and Nutrition, National Institutes of Biomedical Innovation, Health and Nutrition, 1-23-1 Toyama, Shinjuku-ku, Tokyo, 162-8636 Japan; 2 Institute for Active Health, Institute of Interdisciplinary Research, Kyoto University of Advanced Science, Kyoto, 621-8555 Japan; 3 Faculty of Food and Health Sciences, Showa Women’s University, Tokyo, 154-8533 Japan; 4 Physical Fitness Research Institute, Meiji Yasuda Life Foundation of Health and Welfare, Tokyo, 192-0001 Japan; 5 Department of Data Science, Shiga University, Shiga, 522-8522 Japan; 6 Department of Health, Sports and Nutrition, Faculty of Health and Welfare, Kobe Women’s University, Hyogo, 650-0046 Japan; 7 Department of Health and Nutrition, Faculty of Health and Human Life, Nagoya Bunri University, Aichi, 492-8520 Japan; 8 Faculty of Applied Biosciences, Tokyo University of Agriculture, Tokyo, 156-8502 Japan; 9 Laboratory of Applied Health Sciences, Kyoto Prefectural University of Medicine, Kyoto, 602-8566 Japan; 10 Department of Nursing, Doshisha Women’s College of Liberal Arts, Kyoto, 610-0394 Japan; 11 Faculty of Sport Sciences, Waseda University, Saitama, 359-1192 Japan

**Keywords:** Diet quality, Physical frailty, Comprehensive frailty, Japanese Food Guide Spinning Top, Cross-sectional study

## Abstract

**Purpose:**

While the association between diet quality and mortality has been previously demonstrated, the association between frailty and diet quality has not been evaluated well. This study aimed to investigate the association between diet quality and prevalence of both physical and comprehensive frailty, using two validated tools, in a community-based cohort of older adults.

**Methods:**

We conducted cross-sectional analyses using baseline data of 7022 participants aged ≥ 65 years in the Kyoto-Kameoka study. Diet quality was assessed by calculating the adherence scores to the Japanese Food Guide Spinning Top using a validated questionnaire; the participants were stratified into quartile groups based on these scores. Physical and comprehensive frailty was assessed using the Fried phenotype model-based Frailty Screening Index and the Kihon Checklist, respectively. Multivariable logistic regression and the restricted cubic spline model were used to calculate odds ratios (ORs) and their 95% confidence intervals (CIs) for associations between adherence scores and frailty prevalence.

**Results:**

Higher adherence scores signified a higher intake of vitamin C, vegetables, dairy products, and fruits. Physical and comprehensive frailty prevalence was 14.2 and 35.8%, respectively. In a multivariable adjusted model, compared with the bottom adherence score quartile, the top quartile was associated with lower ORs of physical (OR 0.64; 95% CI 0.52–0.80) and comprehensive frailty (OR 0.60; 95% CI 0.51–0.71). These relationships were similar to results in the spline model.

**Conclusions:**

This study shows an inverse dose–response relationship between diet quality and prevalence of both physical and comprehensive frailty in older adults.

**Supplementary Information:**

The online version contains supplementary material available at 10.1007/s00394-022-02819-w.

## Introduction

Frailty is a condition in which multiple physiological systems decline in function due to loss of homeostasis against the stress response [1, 2] and/or increased risk of adverse health outcomes [3] and is characterized by a multidimensional factor such as physical, psychosocial, and cognitive ability playing a part in its development [1–3]. It is an important global public health problem among older adults [3], and it has been reported that frailty is associated with adverse outcomes such as mortality [4, 5], fractures [5, 6], and falls [5]. Therefore, frailty must be prevented to ensure a healthy life expectancy without disease [7] and reduce the burden of healthcare-related costs [8]. Lifestyle care should shift towards more appropriate strategies that prevent frailty [1].

Diet quality is important for promoting health and lowering the risk of major diet-related diseases such as cancer [9–11]. Diet quality is measured using various adherence scores based on previous studies or national dietary guidelines [10, 11]. The use of the Mediterranean diet intake [12–14], the Healthy Eating Index (HEI) [14, 15], or Dietary Approaches to Stop Hypertension (DASH) [14] may decrease the risk or odds ratios (ORs) of frailty. In Japan, the Japanese Food Guide Spinning Top was developed by the Ministry of Health, Labour, and Welfare and the Ministry of Agriculture, Forestry and Fisheries to promote healthy dietary habits [16]. While adherence to this guideline is inversely associated with mortality in middle-aged and older adults [17], it is unclear whether this diet quality is associated with frailty prevalence in older people.

Previous studies have reported an association between diet quality and defined frailty, using specific assessment tools [12–15]. Although the term “frailty” appears straightforward, its evaluation has varied among studies because many such assessment tools exist [18]. This can result in too much heterogeneity in predictive ability and classification. Therefore, measuring the association between diet quality and frailty prevalence needs to be done using a well-validated method. Notably, a previous study demonstrated that disease outcomes in patients admitted to the hospital with COVID-19 [19] were better predicted by frailty than by either age or comorbidity. This underlines the importance of assessing the impact of diet quality on frailty prevalence. Thus, we aimed to investigate the association between diet quality and prevalence of physical and comprehensive frailty using two validated assessment tools.

## Subjects and methods

### Study population and baseline characteristic assessment

The Kyoto-Kameoka study is an ongoing population-based cohort study of community-dwelling residents aged ≥ 65 years in Kameoka City, Japan. Residents were invited to participate in a baseline survey that included the Fried phenotype (FP) model-based Frailty Screening Index (FSI) and the Kihon Checklist (KCL) for the assessment of frailty status. The baseline survey took place on July 29, 2011, and details have been published elsewhere [20–27]. Our group conducted an additional survey, including the administration of the Food Frequency Questionnaire (FFQ), on February 14, 2012. Results of both surveys were collected via postal mail, and informed consent was obtained from participants along with their responses to these questionnaires. Information on medical history, socioeconomic status, health-related lifestyle behaviours, including smoking and drinking status, dietary habits, and physical activity, were obtained on each survey. The study was approved by the Ethics Committees of Kyoto Prefectural University of Medicine (RBMR-E-363), Kyoto University of Advanced Science (No. 20–1), and the National Institutes of Biomedical Innovation, Health and Nutrition (NIBIOHN-76–2). This study has been carried out in accordance with The Code of Ethics of the World Medical Association (Declaration of Helsinki) and Strengthening the Reporting of Observational Studies in Epidemiology (STROBE)-Nutritional Epidemiology criteria [28].

Out of the total number of participants at baseline (*n* = 13,294), 8319 responded to our additional survey. Those with an incomplete KCL or FSI (*n* = 1089), who had self-reported “needed long-term care” (*n* = 122), and who had extremely high or low energy intakes (standard deviation [SD] of 3 from the mean energy intake) [23] in each sex group (*n* = 86), were excluded. Ultimately, 7022 participants were included in the present analysis.

### Dietary assessment

Dietary nutrient and food intakes were estimated using the FFQ [24, 25, 29–31]. We have previously validated the FFQ using dietary records [24, 29, 31] and doubly labelled water (DLW) methods [25, 32] in a subpopulation of participants of the Kyoto-Kameoka study. On the FFQ, participants were asked to report their intake frequency of 47 food and beverage items over the past year. Food and nutrient intakes were then calculated using the frequency of appearance of each food and beverage category and the portion size for each sex group [30] or using a program based on *the Standard Tables of Food Composition in Japan* [33]. Energy intake was calibrated using our previously developed equation that uses total energy expenditure (as measured by the DLW method) [25]. The equation used for this calculation attenuates the impact of age, sex, and body mass index (BMI)-related self-reporting bias on estimated energy intake in older adults, assessed via the FFQ [32].

### Calculation of diet quality score

To assess diet quality, we calculated participants’ adherence scores from the Japanese Food Guide Spinning Top (the food-based Japanese dietary guidelines) [16]. The details of this measure have been published elsewhere [16, 17, 27, 34]. Recommended serving amounts for each category and recommended total energy intake are specified according to sex, age, and level of physical activity (i.e., participation in manual labour or walking for at least 1 h a day were labelled as moderately physically active; other activity levels were labelled as sedentary) [16, 17, 27, 34]. We calculated the number of servings according to the food guide criteria. One serving of each was defined as follows: a grain dish (40 g of carbohydrates), vegetable dish (weight, 70 g), fish or meat dish (6 g of protein), milk (100 mg calcium), and fruit (weight of 100 g) [16, 17, 27, 34]. We determined food guide adherence scores using a method described by Kurotani et al. [17, 27]. To calculate the scores for each component, food items were categorised as follows: grain dishes (rice, bread, and noodles); vegetable dishes (potatoes, pumpkin/squash, carrots, broccoli, green-leaved vegetables, other green/yellow vegetables, cabbage, daikon (Japanese radish), kiriboshi-daikon (dry strips of Japanese radish), burdock, bamboo shoots, other vegetables, mushrooms, seaweed, peanuts, and almonds); fish and meat dishes (tofu (soybean curd), natto (fermented soybeans), soybeans, eggs, chicken, beef, pork, liver, ham, sausage, bacon, salami sausage, fish, bone-edible small fish, canned tuna, cuttlefish, squid, octopus, shrimp, crab, shellfish, fish eggs, fish paste products, ganmodoki (fried tofu paste), and nama-age (fried tofu); [soybeans were included in the fish and meat dish category, based on their high-protein nutrient profile]); milk products (milk and yogurt); fruits (citrus and other fruits); and snacks and alcoholic beverages (western-style confectioneries, Japanese-style confectioneries, and alcohol). In addition to the Japanese Food Guide Spinning Top criteria, we calculated adherence scores based on the intake ratio of white to red meat as a component of the score [17, 27]. White meat included chicken, fish, bone-edible small fish, canned tuna, cuttlefish, squid, octopus, shrimp, crab, shellfish, fish eggs, and fish paste products. Red meat included beef, pork, liver, ham, sausage, bacon, and salami sausage. Each component was given a score between 0 and 10, and to obtain a total score that ranged from 0 (lowest adherence) to 80 (highest adherence), all the component scores were summed.

### Definition of frailty

Frailty was assessed using the FP model-based five-item FSI [7, 21–23] and the 25-item KCL [21–23, 35, 36], both of which have been validated according to the risk of Long-Term Care Insurance Certification [7, 35]. Physical frailty was evaluated using the FSI, focusing on the physical aspects, and was defined as a score of 3 or more points out of a maximum of 5 (weight loss, slow gait speed, cognition, exhaustion, and low physical activity) [7, 21–23]. Comprehensive frailty was evaluated using the KCL. It includes cognitive and social aspects, as well as physical factors, and was defined as a score of 7 or higher out of a possible 25 points [21–23, 35, 36]. Additionally, we investigated possible associations between the FSI and KCL subdomains and the adherence score. The KCL consists of seven subdomains: instrumental activity of daily living (IADL) disability, physical, nutrition, oral, social, cognitive, and depression. Detailed methods of the subdomain investigation are provided elsewhere [26].

### Statistical analysis

Adherence scores to the Japanese Food Guide Spinning Top were divided into quartiles (Qs). Continuous and categorical variables are shown as means with SDs and numbers with percentages, respectively. Where information pertaining to the BMI (*n* = 35), alcohol status (*n* = 102), smoking status (*n* = 385), physical activity (*n* = 359), family structure (*n* = 468), socioeconomic status (*n* = 235), education attainment (*n* = 645), denture use (*n* = 82), or medications (*n* = 387) was missing, we performed multiple imputation on five data sets, using chained equations from R statistical software [37] to prevent a systematic error appearing due to selection bias. Missing data were assumed to be missing at random. Nutrient intake was adjusted for energy intake per 1000 kcal via the nutrient density method, using uncalibrated energy intake [38]. These values are shown as the median of each quartile group. The association between adherence score and nutrient intake was confirmed using Spearman’s correlation analysis.

The number of frailty cases is shown as a case (%). Multivariate logistic regression analysis was performed, considering several baseline covariate variables. Multivariate adjusted analyses were verified by two models: Model 1 was adjusted for age (continuous), sex (female or male), and population density (≥ 1000 or < 1000 people/km^2^). In model 2, in addition to the factors adjusted for in model 1, we adjusted for body mass index (continuous), physical activity (yes or no), denture use (yes or no), smoking status (never smoker, past smoker, or current smoker), alcohol intake status (every day, sometimes, seldom, or never), educational attainment (< 9, 10–12, or ≥ 13 years), medication use (continuous), living alone (yes or no), socioeconomic status (high or low), green tea consumption (frequency), coffee consumption (frequency), and history of the disease (hypertension, diabetes, dyslipidaemia, heart disease, and stroke; yes or no). These variables were selected according to covariates used in a previous study [21–23, 26]. ORs for frailty are presented as OR (95% confidence interval [CI]), using the first quartile group (lowest diet quality) as the referent group. Diet quality is better among women [17] and affluent people [39]. Thus there is a possibility of evaluating primarily sex or socioeconomic status-specific influences when ranking individuals according to adherence scores. Therefore, subgroup analyses were performed separately by sex and socioeconomic status (high or low). Additionally, we evaluated possible associations between the adherence score and the subdomains that were evaluated using the FSI and KCL.

To evaluate the curve association of diet quality and frailty prevalence, we used a restricted cubic spline model considering three data points (5th, 50th, and 95th percentiles), based on the distribution of the recommended adherence score. Because the data were sparse, we truncated the analysis at 29 points (1% of the distribution) [22]. We calculated the ORs for frailty prevalence associated with adherence score, using the 43 points of first quartile value as the reference in the restricted cubic spline model [21]. Moreover, we considered the association between frailty prevalence and the adherence score of each component of the Japanese Food Guide Spinning Top. A two-sided *p* value < 0.05 was considered significant. Linear trends were computed by treating adherence score exposure as a continuous variable. Statistical analyses were performed using STATA MP, version 15.0 (Stata Corp LP, College Station, TX), and/or R software 3.4.3 (R Core Team, Vienna, Austria).

## Results

The mean score on the adherence to the Japanese Food Guide Spinning Top was 54.3 (SD 8.4). Compared to participants with lower adherence scores, participants with higher scores were likelier to be women, live alone, have higher educational attainment and socioeconomic status, and have a history of hyperlipidaemia. They were less likely to be current smokers and alcohol drinkers (Table 1).

**Table 1:** Baseline participant characteristics according to quartile of adherence to the Japanese Food Guide Spinning Top

	Total (*n* = 7022)		Quartile of the Japanese Food Guide Spinning Top score								*p* value
			Q1 (*n* = 1756)		Q2 (*n* = 1756)		Q3 (*n* = 1755)		Q4 (*n* = 1755)		
Age [years]^a^	73.3	(6.1)	73.5	(6.2)	73.2	(6.2)	73.0	(6.0)	73.4	(6.0)	**0.039**
Women [*n* (%)]^b^	3709	(52.8)	551	(31.4)	874	(49.8)	1044	(59.5)	1240	(70.7)	**< 0.001**
PD ≥ 1000 people/km^2^ [*n* (%)]^b^	3232	(46.0)	738	(42.0)	781	(44.5)	814	(46.4)	899	(51.2)	**< 0.001**
BMI [kg/m^2^]^a^	22.6	(3.0)	22.7	(3.1)	22.7	(3.1)	22.7	(3.0)	22.3	(2.9)	**< 0.001**
Alcohol drinker [*n* (%)]^b^	4595	(65.4)	1309	(74.5)	1174	(66.9)	1077	(61.4)	1035	(59.0)	**< 0.001**
Current smoker [*n* (%)]^b^	754	(10.7)	335	(19.1)	212	(12.1)	115	(6.6)	92	(5.3)	**< 0.001**
MVPA [*n* (%)]^b^	3114	(44.4)	746	(42.5)	802	(45.7)	788	(44.9)	778	(44.3)	0.268
Living alone [*n* (%)]^b^	789	(11.2)	171	(9.7)	167	(9.5)	214	(12.2)	237	(13.5)	**< 0.001**
HSES [*n* (%)]^b^	2394	(34.1)	507	(28.9)	546	(31.1)	623	(35.5)	718	(40.9)	**< 0.001**
Education ≥ 13 y [*n* (%)]^b^	1576	(22.4)	359	(20.4)	386	(22.0)	414	(23.6)	417	(23.8)	**< 0.001**
Denture use [*n* (%)]^b^	4212	(60.0)	1099	(62.6)	1074	(61.2)	1015	(57.8)	1024	(58.4)	**0.010**
No medication [*n* (%)]^b^	1575	(22.4)	405	(23.1)	431	(24.5)	367	(20.9)	372	(21.2)	**< 0.001**
Hypertension [*n* (%)]^b^	2731	(38.9)	656	(37.4)	677	(38.6)	709	(40.4)	689	(39.3)	0.308
Stroke [*n* (%)]^b^	251	(3.6)	71	(4.0)	78	(4.4)	54	(3.1)	48	(2.7)	**0.020**
Heart disease [*n* (%)]^b^	869	(12.4)	247	(14.1)	228	(13.0)	175	(10.0)	219	(12.5)	**0.002**
Diabetes [*n* (%)]^b^	736	(10.5)	193	(11.0)	190	(10.8)	180	(10.3)	173	(9.9)	0.677
Hyperlipidemia [*n* (%)]^b^	711	(10.1)	112	(6.4)	150	(8.5)	193	(11.0)	256	(14.6)	**< 0.001**

Bold *p* values are statistically significant (*p* < 0.05). Data of participants with missing values underwent multiple imputation: body mass index (*n* = 35); alcohol status (*n* = 102); smoking status (*n* = 385); physical activity (*n* = 359); family structure (*n* = 468); socioeconomic status (*n* = 235); education attainment (*n* = 645); denture use (*n* = 82); medications (*n* = 387). BMI, body mass index; HSES, high socioeconomic status; MVPA, moderate to vigorous physical activity; PD, population density. Q1 through Q4 includes Japanese Food Guide Spinning Top scores of < 49.5, 49.5–54.8, 54.9–60.1, and ≥ 60.2

^a^Continuous values are shown as mean (standard deviation) and are analyzed using variance analysis

^b^Categorical values are shown as number (percentage) and are analyzed using the Chi-square test. MVPA means reported MVPA exercise habits from a questionnaire

The associations between adherence scores and nutrient and food intake are shown in Table 2. Adherence scores were moderately correlated to the intake of the following: vitamin C (*r* = 0.46), vegetables (*r* = 0.42), fruits (*r* = 0.56), and dairy products (*r* = 0.46). Participants with higher adherence scores tended to have a higher intake of fat, folate, and calcium (≥ *r* = 0.30). Therefore, participants with higher adherence scores have characteristics of high vitamin C, vegetables, fruits, and dairy products intake. However, energy, protein, and carbohydrate intake were not observed to be significantly different.

**Table 2:** Association between energy and nutrient intake and adherence to the Japanese Food Guide Spinning Top

	Quartile of the Japanese Food Guide spinning top score				*r*^a^
	Q1 (*n* = 1756)	Q2 (*n* = 1756)	Q3 (*n* = 1755)	Q4 (*n* = 1755)	
Nutrients					
Calibrated EI [kcal/day]^b^	2294	2151	2047	1995	− 0.23
Uncalibrated EI [kcal/day]	1832	1722	1702	1697	− 0.05
Protein [% energy/day]	10.7	12.0	12.4	12.7	0.28
Fat [% energy/day]	19.1	23.3	25.9	28.7	0.35
Carbohydrate [% energy/day]	55.9	56.0	55.7	55.6	− 0.01
SFA [g/1000 kcal/day]	5.4	6.4	6.9	7.4	0.26
MUFA [g/1000 kcal/day]	8.2	9.6	10.7	12.0	0.29
PUFA [g/1000 kcal/day]	7.0	8.3	8.9	9.8	0.27
n-6 PUFA [g/1000 kcal/day]	6.0	7.3	7.8	8.7	0.28
n-3 PUFA [g/1000 kcal/day]	1.0	1.2	1.3	1.5	0.29
Cholesterol [mg/1000 kcal/day]	108	119	122	125	0.12
Vitamin A [µg RE/1000 kcal/day]^c^	393	477	520	492	0.11
Vitamin D [µg/1000 kcal/day]	2.3	2.5	2.5	2.4	0.03
α-Tocopherol [mg/1000 kcal/day]	4.5	5.4	5.8	6.4	0.28
Folate [µg/1000 kcal/day]	143	174	191	216	0.34
Vitamin C [mg/1000 kcal/day]	41	52	61	75	0.46
Sodium [mg/1000 kcal/day]	897	972	978	981	0.08
Potassium [mg/1000 kcal/day]	978	1101	1161	1226	0.29
Iron [mg/1000 kcal/day]	3.2	3.7	3.8	4.0	0.19
Calcium [mg/1000 kcal/day]	224	271	309	331	0.31
Total dietary fiber [g/1000 kcal/day]	5.0	5.8	6.1	6.6	0.28
Alcohol [g/1000 kcal/day]	0.8	0.0	0.0	0.0	− 0.22
Food					
Grains [g/1000 kcal/day]	272	262	253	247	− 0.15
Vegetables [g/1000 kcal/day]	59	85	99	126	0.42
Fruits [g/1000 kcal/day]	13	25	44	70	0.56
Dairies [g/1000 kcal/day]	6	42	81	95	0.46
Red meats [g/1000 kcal/day]	11	12	12	9	− 0.03
White meats [g/1000 kcal/day]	34	44	46	47	0.19
Confectionery [g/1000 kcal/day]	10	9	9	8	− 0.13

All values are shown medians or correlation coefficients. Nutrient intake was adjusted for energy intake via the nutrient density method, using uncalibrated energy intake. Values are shown as medians in each quartile group. Q1 through Q4 included Japanese Food Guide Spinning Top scores of < 49.5, 49.5–54.8, 54.9–60.1, and ≥ 60.2. EI, energy intake; MUFA, monounsaturated fatty acid; PUFA, polyunsaturated fatty acid; SFA, saturated fatty acid

^a^Spearman’s correlation analysis was used to evaluate the relationship between nutrient intake and adherence score

^b^Calibrated EI was calculated using an equation developed by the authors

^c^Sum of retinol, β-carotene/12, α-carotene/24, and cryptoxanthin/24

Table 3 shows the associations between adherence score and physical and comprehensive frailty prevalence. Physical and comprehensive frailty prevalence was 14.2 and 35.8%, respectively. We found that adherence score was negatively associated with the OR of prevalence of physical frailty, as defined by the FP model, after adjusting for baseline potential confounding factors (Q1: reference; Q2: OR 0.87; 95% CI 0.72–1.06; Q3: OR 0.84; 95% CI 0.69–1.03; Q4: OR 0.64; 95% CI 0.52–0.80); *P* for trend < 0.001). Similar findings were observed with comprehensive frailty. In subgroup analyses, comparable results were observed in both the sex and socioeconomic status-stratified models (Supplemental Tables 1 and 2). Furthermore, we demonstrated that the adherence score was negatively associated with ORs for the prevalence of slow gait speed, cognition, exhaustion, and low physical activity, after evaluation using the FP model subdomains (Supplementary Table S3). These indicated a negative relationship with ORs for the prevalence of IADL disability, social frailty, cognitive frailty, and depression after evaluation using the KCL subdomains (Supplementary Table S4).

**Table 3:** Multivariable adjusted odds ratios and 95% confidence intervals of the prevalence of physical and comprehensive frailty according to Japanese Food Guide Spinning Top adherence score

	Quartile of the Japanese Food Guide Spinning Top score								10 points increment		*p* for trend^a^
	Q1 (*n* = 1756)		Q2 (*n* = 1756)		Q3 (*n* = 1755)		Q4 (*n* = 1755)				
Mean (SD) score	43.2	(5.9)	52.3	(1.6)	57.5	(1.5)	64.1	(3.0)			
Physical frailty											
Case [*n* (%)]	302	(17.2)	259	(14.7)	241	(13.7)	196	(11.2)			
Model 1^b^	1.00	(Ref)	0.81	(0.68–0.98)	0.75	(0.62–0.90)	0.56	(0.45–0.68)	0.79	(0.71–0.87)	**< 0.001**
Model 2^c^	1.00	(Ref)	0.87	(0.72–1.06)	0.84	(0.69–1.03)	0.64	(0.52–0.80)	0.85	(0.76–0.93)	**< 0.001**
Comprehensive frailty											
Case [*n* (%)]	744	(42.4)	658	(37.5)	593	(33.8)	519	(29.6)			
Model 1^b^	1.00	(Ref)	0.79	(0.68–0.91)	0.66	(0.57–0.76)	0.49	(0.42–0.57)	0.70	(0.64–0.76)	**< 0.001**
Model 2^c^	1.00	(Ref)	0.86	(0.73–1.00)	0.77	(0.66–0.90)	0.60	(0.51–0.71)	0.79	(0.72–0.86)	**< 0.001**

All values are shown means (SDs), numbers (%), or relative ORs (95% CI). All estimates were derived from a multivariable logistic regression model. Physical and comprehensive frailty was assessed using the validated Fried phenotype model-based Frailty Screening Index and the Kihon Checklist. Bold *p* values are statistically significant (*p* < 0.05). Q1 through Q4 included Japanese Food Guide Spinning Top scores of < 49.5, 49.5–54.8, 54.9–60.1, and ≥ 60.2

CI, confidence interval; OR, odds ratio; Ref, reference; SD, standard deviation

^a^Linear trend *p* values were calculated with the likelihood ratio test using continuous variables of adherence scores

^b^Model 1 was adjusted for age (continuous), sex (female or male), and population density (≥ 1000 or < 1000 people/km^2^)

^c^Model 2 was Model 1 with mutual adjustments for body mass index (continuous), physical activity (yes or no), denture use (yes or no), smoking status (never smoker, past smoker, and current smoker), alcohol intake status (every day, sometimes, seldom, or never), educational attainment (< 9, 10–12, or ≥ 13 years), medication use (continuous), living alone (yes or no), socioeconomic status (high or low), green tea consumption (frequency), coffee consumption (frequency), and history of disease (hypertension, diabetes, dyslipidaemia, heart disease, and stroke; yes or no)

**Fig. 1:**
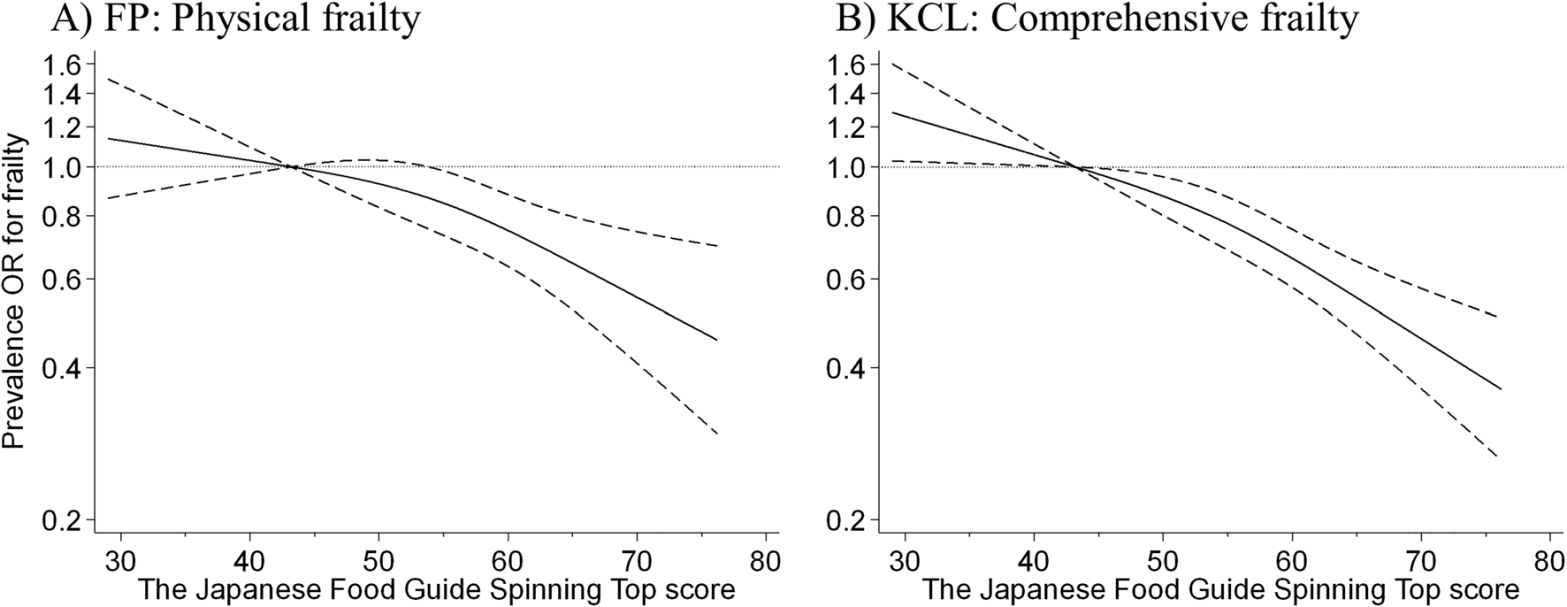
Relationship between the Japanese Food Guide Spinning Top adherence score and frailty, based on **A** fried phenotype (FP) model and **B** the Kihon Checklist (KCL), using a restricted cubic spline logistic regression model. Frailty, according to the FP model-based self-administered frailty screening index (FSI), was defined as a score of ≥ 3 out of 5 points. Frailty, according to the KCL, was defined as a score ≥ 7 out of 25 points. Solid lines represent odds ratios (ORs), and dashed lines represent 95% confidence intervals (CIs). We calculated ORs for frailty prevalence using a first quartile value of 43 points as the reference. This analysis included 6954 participants. We estimated that *p* < 0.05 when 95% CI of the OR did not exceed 1.00, and *p* ≥ 0.05 when 95% CI of the OR exceeded 1.00. Analyses were adjusted for age, sex, population density, body mass index, physical activity, denture use, smoking status, alcohol intake status, educational attainment, medication use, living alone, socioeconomic status, green tea consumption, coffee consumption, and history of disease (hypertension, diabetes, dyslipidaemia, heart disease, and stroke)

To evaluate the curve of the association between frailty prevalence and adherence score, we used a restricted cubic spline model (Fig. 1). When the first quartile (43 points) for adherence score was used as the reference, the shape of the curve showed a modest and negative association between adherence score and prevalence of each type of frailty, defined by the FP model or the KCL, up to approximately 55 points. This is the average adherence score of the current study population. Beyond this, an adherence score of up to approximately 76 points showed a strong dose-dependent negative association with frailty prevalence.

The association of the adherence score of each component with physical frailty prevalence is shown in Table 4. After adjusting for confounders, the top adherence score quartile revealed a significant association with a lower OR of physical frailty (reference, lowest quartile of adherence scores): vegetable dishes (OR 0.86 [95% CI 0.70–1.06], adjusted *p* for trend = 0.041) and milk (OR 0.79 [95% CI 0.65–0.97], adjusted *p* for trend = 0.003). Similar findings were observed with comprehensive frailty as defined by the KCL (Supplemental Tables 5). Furthermore, a high score on adherence to fruits and snacks and alcoholic beverages was associated with a low prevalence of comprehensive frailty, while a high score on adherence to energy intake showed a positive relationship with the prevalence of comprehensive frailty.

**Table 4:** Multivariable adjusted odds ratios and 95% confidence intervals of the prevalence of physical frailty according to adherence score of each component on the Japanese Food Guide Spinning Top

	Quartile of the Japanese Food Guide Spinning Top score								1 points increment		*p* for trend^a^
	Q1 (*n* = 1756)		Q2 (*n* = 1756)		Q3 (*n* = 1755)		Q4 (*n* = 1755)				
Grain dishes											
Case [*n* (%)]	250	(14.2)	257	(14.6)	243	(13.8)	248	(14.1)			
Model 1^b^	1.00	(Ref)	1.01	(0.84 to 1.23)	0.92	(0.76–1.11)	0.88	(0.73–1.07)	0.97	(0.95–1.00)	0.079
Model 2^c^	1.00	(Ref)	1.02	(0.84–1.24)	0.92	(0.76–1.12)	0.86	(0.70–1.04)	0.98	(0.95–1.00)	0.098
Vegetable dishes											
Case [*n* (%)]	273	(15.5)	265	(15.1)	231	(13.2)	229	(13.0)			
Model 1^b^	1.00	(Ref)	0.97	(0.81–1.17)	0.79	(0.65–0.96)	0.73	(0.60–0.89)	0.95	(0.92–0.97)	**< 0.001**
Model 2^c^	1.00	(Ref)	1.03	(0.85–1.25)	0.87	(0.71–1.07)	0.86	(0.70–1.06)	0.97	(0.95–0.99)	**0.041**
Fish and meat dishes											
Case [*n* (%)]	269	(15.3)	266	(15.1)	225	(12.8)	238	(13.6)			
Model 1^b^	1.00	(Ref)	1.07	(0.89–1.29)	0.92	(0.76–1.12)	0.99	(0.82–1.20)	1.00	(0.98–1.02)	0.801
Model 2^c^	1.00	(Ref)	1.02	(0.85–1.24)	0.87	(0.71–1.06)	0.95	(0.78–1.15)	0.99	(0.97–1.01)	0.294
Milk											
Case [*n* (%)]	307	(17.5)	264	(15.0)	204	(11.6)	223	(12.7)			
Model 1^b^	1.00	(Ref)	0.90	(0.75–1.09)	0.66	(0.54–0.80)	0.71	(0.59–0.86)	0.96	(0.94–0.98)	**< 0.001**
Model 2^c^	1.00	(Ref)	0.92	(0.77–1.11)	0.71	(0.58–0.86)	0.79	(0.65–0.97)	0.97	(0.95–0.99)	**0.003**
Fruits											
Case [*n* (%)]	300	(17.1)	246	(14.0)	221	(12.6)	231	(13.2)			
Model 1^b^	1.00	(Ref)	0.79	(0.65–0.95)	0.67	(0.55–0.81)	0.68	(0.56–0.83)	0.96	(0.93–0.98)	**< 0.001**
Model 2^c^	1.00	(Ref)	0.87	(0.72–1.06)	0.78	(0.64–0.95)	0.83	(0.68–1.02)	0.98	(0.96–1.00)	0.109
Total energy											
Case [*n* (%)]	230	(13.1)	243	(13.8)	278	(15.8)	247	(14.1)			
Model 1^b^	1.00	(Ref)	1.31	(1.05–1.62)	1.44	(1.14–1.82)	1.17	(0.91–1.51)	1.16	(1.05–1.29)	**0.004**
Model 2^c^	1.00	(Ref)	1.19	(0.95–1.48)	1.19	(0.93–1.53)	1.07	(0.82–1.39)	1.09	(0.98–1.22)	0.108
Snacks and alcohol											
Case [*n* (%)]	270	(15.4)	263	(15.0)	234	(13.3)	231	(13.2)			
Model 1^b^	1.00	(Ref)	0.97	(0.80–1.17)	0.81	(0.66–0.98)	0.77	(0.63–0.95)	1.00	(0.96–1.04)	0.945
Model 2^c^	1.00	(Ref)	0.94	(0.77–1.14)	0.84	(0.68–1.02)	0.86	(0.70–1.06)	0.99	(0.96–1.03)	0.655
White–red meat											
Case [*n* (%)]	262	(14.9)	242	(13.8)	276	(15.7)	218	(12.4)			
Model 1^b^	1.00	(Ref)	0.94	(0.78–1.14)	1.05	(0.87–1.26)	0.76	(0.63–0.93)	0.99	(0.96–1.01)	0.271
Model 2^c^	1.00	(Ref)	0.96	(0.79–1.17)	1.02	(0.84–1.23)	0.81	(0.66–0.99)	0.99	(0.96–1.01)	0.389

All values are shown numbers (%), or relative ORs (95% CI). All estimates were derived from a multivariable logistic regression model. Bold *p* values are statistically significant (*p* < 0.05). The range of Q1 through Q4 for each food score is shown following; < 7.1, 7.1–8.9, 9.0–9.8, and ≥ 9.9 score for grain dishes, < 2.7, 2.7–4.3, 4.4–6.4, and ≥ 6.5 score for vegetable dishes, < 4.8, 4.8–7.7, 7.8–9.7, and ≥ 9.8 score for fish and meat dishes, < 0.6, 0.6–5.2, 5.3–7.7, and ≥ 7.8 score for milk, < 1.3, 1.3–3.1, 3.2–5.5, and ≥ 5.6 score for fruits, < 8.8, 8.8–9.3, 9.4–9.8, and ≥ 9.9 score for total energy intake, < 8.7, 8.7–9.5, 9.6–9.8, and ≥ 9.9 score for snacks and alcohol, and < 5.8, 5.8–8.9, 9.0–9.8, and ≥ 9.9 score for white to red meat

CI, confidence interval; OR, odds ratio; Ref, reference

^a^Linear trend *p* values were calculated with the likelihood ratio test using the continuous variables of adherence scores

^b^Model 1 was adjusted for age (continuous), sex (female or male), and population density (≥ 1000 or < 1000 people/km^2^)

^c^Model 2 was Model 1 with mutual adjustment for body mass index (continuous), physical activity (yes or no), denture use (yes or no), smoking status (never smoker, past smoker, and current smoker), alcohol intake status (every day, sometimes, seldom, or never), educational attainment (< 9, 10–12, or ≥ 13 years), medication use (continuous), living alone (yes or no), socioeconomic status (high or low), green tea consumption (frequency), coffee consumption (frequency), and history of disease (hypertension, diabetes, dyslipidaemia, heart disease, and stroke; yes or no)

## Discussion

Our study showed an inverse dose–response relationship between scores on adherence to the Japanese Food Guide Spinning Top and the prevalences of physical and comprehensive frailty. Higher scores of adherence to vegetable dishes and milk intake were inversely associated with both frailty types. To the best of our knowledge, this is the first study to examine the association between diet quality and prevalence of both physical and comprehensive frailty in older adults, defined by two different validation methods. Our results may corroborate the essential role of adherence to dietary guidelines in individual health.

To evaluate physical and comprehensive frailty, we used two different methods: an FP model and the KCL. The prevalence of comprehensive frailty defined by the KCL (35.8%) was significantly higher than that defined by the FP model (14.2%). This is consistent with previous findings [20, 36]. The people identified as physical and comprehensive frailty may not necessarily match [40]. Previous studies comparing comprehensive frailty, which considers multiple factors, and physical frailty, which focuses on physical factors only, reported that comprehensive frailty had higher accuracy in predicting the risk of mortality [41, 42]. The reason for this is that comprehensive frailty indices that assess frailty using a multi-faceted model have a positive linear correlation with age and thus reflect biological ageing [43]. Therefore, it is also needed to evaluate the association between diet quality, physical frailty, and comprehensive frailty.

In our study, higher scores on adherence were inversely associated with both physical and comprehensive frailty prevalence after adjustment for potential confounding variables. Previous studies on associations between diet quality and frailty focused mainly on the physical components [12, 13, 15]. Our findings were consistent across both types of frailty, despite several differences in indices. Despite differences in dietary measures, our findings, taken together with those from previous studies, suggest that a healthy dietary pattern is associated with a lower OR of frailty. Moreover, we also observed an inverse association between adherence scores and ORs of subdomains in the FP model and KCL. Given that the risk of the incidence of dementia and depression tends to be higher in older adults [44], it is necessary to consider the association between diet quality and physical frailty and the risk factors for comprehensive frailty. Therefore, our results may help shed more light on key dietary components in further investigations into untangling the relationship between diet and frailty.

Although it is unclear the detail of mechanism that diet quality is inversely associated with frailty prevalence, two reasons can be considered from the previous studies. The first is that some nutrient intake is negatively associated with the risk of frailty [45, 46]. Our results indicate a positive correlation between scores on adherence and vitamin C. Actually, a previous prospective cohort study has reported that low vitamin C intake is associated with the risk of frailty in Spanish older adults [46]. The second is that people with high diet quality maintain better nutritional status. Many researchers have considered that healthy dietary pattern is inversely associated with the risk of the double burden of malnutrition, such as underweight and obesity [47]. We have previously reported that the lowest prevalence of both physical and comprehensive frailty was found at approximately 40 kcal/kg IBW/day (ideal body weight = 22 × height^2^) with DLW-calibrated energy intake [23] and BMI 21.4–25.7 kg/m^2^ [22]. Such facts should support our data, showing that frailty prevalence is lower among people with high diet quality. These previous studies may support our results. Although there are multiple mechanisms, such as antioxidant effects and metabolic and cardiovascular benefits, through which a healthy diet may contribute to a lower risk of frailty [14], causal relationships and detailed mechanisms must be clarified with further basic and intervention studies.

A strength of this study is that it provides additional evidence of the association between diet quality and physical and comprehensive frailty, using two validated assessment tools. These results indicate a potential robust association between diet quality and frailty. However, some methodologic limitations should be mentioned. First, our study utilised a cross-sectional study design. Therefore, no temporal or direct causal relationships of the observed associations between diet quality and frailty prevalence can be inferred. Second, although we have used previously validated FFQ [24, 25, 29–31], given that the adherence scores to the Japanese Food Guide Spinning Top are based on specific absolute cut-offs for dietary components, it may be insufficient to evaluate the overall diet quality because we evaluated it using the FFQ, consisting of 47 food and beverage items. However, we have previously reported that the adherence scores to the Japanese Food Guide Spinning Top estimated from our used FFQ are inversely associated with the prevalence of poor oral health-related quality of life [27]. Third, owing to the observational nature of the study, we could not fully exclude residual confounding factors from self-report or unmeasured variables. For example, women and individuals with higher socioeconomic status may have greater opportunities to consume fruits, vegetables, and dairy products [48]. In the sex and socioeconomic status-stratified models, we observed an inverse association between diet quality and the prevalence of comprehensive frailty across all groups (Supplemental Tables 1 and 2). Therefore, the inverse association between diet quality and frailty could not be fully explained by only socioeconomic status or sex alone. Finally, our study included individuals with a history of disease, including hypertension, diabetes, and dyslipidaemia. Extrapolation to participants with attributes other than those included in the study should be performed with caution because such individuals may have received dietary advice for their disease and modified their diets accordingly. However, our results were similar after excluding participants with such diseases. Considering these limitations, rigorous longitudinal studies that further evaluate whether a healthier diet is a modifiable risk factor for frailty are needed.

In recent years, the degree of frailty increased across most adult age groups in the United States between 1999 and 2018 [49]. Our findings indicated that overall adherence scores are strongly negatively associated with ORs of frailty than any individual dietary component in the food-based Japanese dietary guidelines, and these results were similar to the previous study that reported an association between adherence to the Mediterranean diet and mortality [50]. Therefore, our results may provide useful insights for improving and preventing frailty by adherence to the food-based Japanese dietary guidelines in older adults.

In conclusion, the results of this study indicate that higher diet quality with strong adherence to the Japanese Food Guide Spinning Top is associated with a lower prevalence of both physical and comprehensive frailty. The results also show a dose–response relationship between better diet quality and a lower frailty prevalence among community-dwelling older adults. Given the rapid increase in frailty prevalence worldwide, these findings may be encouraging to older adults, who are a high-risk population for frailty.

## Supplementary Information

Below is the link to the electronic supplementary material.

Supplementary file1 (PDF 363 KB)

## Data Availability

Researchers can request our study group for permission to use these data by contacting Y.Y. (yamaday@nibiohn.go.jp).
