# Supplementary material for: Diet quality and physical or comprehensive frailty among older adults
Source: Eur J Nutr. 2022 Feb 13;61(5):2451–62. doi: 10.1007/s00394-022-02819-w (PMC8852878; doi:10.1007/s00394-022-02819-w)
Supplement: Supplementary file 1 — Supplementary file1 (PDF 363 KB) [file 394_2022_2819_MOESM1_ESM.pdf]

Supplementary Material

"Diet quality and physical or comprehensive frailty among older adults"

Authors: Daiki Watanabe, Kayo Kurotani, Tsukasa Yoshida, Hinako Nanri, Yuya Watanabe, Heiwa Date, Aya Itoi, Chiho Goto, Kazuko Ishikawa-Takata, Misaka Kimura, Yosuke Yamada, and Kyoto-Kameoka Study Group

SUPPLEMENTARY TABLES

Supplementary Table 1. Odds ratios for the Japanese Food Guide Spinning Top adherence scores and prevalence of physical frailty defined by the FP model, calculated using sex and socioeconomic status stratified multivariate logistic regression

Supplementary Table 2. Odds ratios for the Japanese Food Guide Spinning Top adherence score and prevalence of comprehensive frailty defined by the Kihon Checklist, calculated using sex and socioeconomic status stratified multivariate logistic regression

Supplementary Table 3. Odds ratios for the Japanese Food Guide Spinning Top adherence score and prevalence of the Fried phenotype model subdomains, calculated using multivariate logistic regression

Supplementary Table 4. Odds ratios for the Japanese Food Guide Spinning Top adherence score and the prevalence of the Kihon Checklist subdomains, calculated using multivariate logistic regression

Supplementary Table 5. Multivariable adjusted odds ratios and 95% confidence intervals of the prevalence of comprehensive frailty according to the adherence score of each component in the Japanese Food Guide Spinning Top

Supplementary Table 1. Odds ratios for the Japanese Food Guide Spinning Top adherence scores and prevalence of physical frailty defined by the FP model, calculated using sex and socioeconomic status stratified multivariate logistic regression [a]

	Quartile of the Japanese food guide Spinning Top score								10 points increment		*p* for trend [b]
	Q1		Q2		Q3		Q4				
Women, n	928		927		927		927				
Mean (SD) score	46.5	(6.2)	54.7	(1.4)	59.5	(1.4)	65.5	(2.8)			
Case [*n* (%)]	180	(19.4)	144	(15.5)	136	(14.7)	98	(10.6)			
Model 1 [c,d]	1.00	(Ref)	0.79	(0.62 to 1.01)	0.75	(0.58 to 0.96)	0.51	(0.39 to 0.67)	0.76	(0.65 to 0.87)	**<0.001**
Model 2 [d,e]	1.00	(Ref)	0.85	(0.66 to 1.09)	0.83	(0.64 to 1.08)	0.58	(0.44 to 0.76)	0.81	(0.69 to 0.92)	**0.001**
Men, n	829		828		828		828				
Mean (SD) score	40.9	(5.5)	49.6	(1.6)	54.9	(1.5)	61.8	(3.2)			
Case [*n* (%)]	137	(16.5)	121	(14.6)	93	(11.2)	89	(10.8)			
Model 1 [c,d]	1.00	(Ref)	0.89	(0.68 to 1.17)	0.66	(0.49 to 0.88)	0.63	(0.47 to 0.84)	0.84	(0.72 to 0.96)	**0.007**
Model 2 [d,e]	1.00	(Ref)	0.98	(0.74 to 1.29)	0.74	(0.55 to 0.99)	0.72	(0.53 to 0.98)	0.90	(0.81 to 0.99)	**0.035**
HSES, n	599		599		598		598				
Mean (SD) score	44.3	(6.0)	53.5	(1.6)	58.7	(1.5)	64.8	(2.8)			
Case [*n* (%)]	76	(12.7)	63	(10.5)	55	(9.2)	54	(9.0)			
Model 1 [c,d]	1.00	(Ref)	0.83	(0.58 to 1.20)	0.70	(0.48 to 1.02)	0.65	(0.44 to 0.96)	0.79	(0.63 to 0.95)	**0.009**
Model 2 [d,e]	1.00	(Ref)	0.94	(0.65 to 1.36)	0.76	(0.51 to 1.13)	0.71	(0.48 to 1.06)	0.81	(0.65 to 0.97)	**0.025**
LSES, n	1,157		1,157		1,157		1,157				
Mean (SD) score	42.7	(5.9)	51.8	(1.5)	56.9	(1.5)	63.7	(3.2)			
Case [*n* (%)]	226	(19.5)	186	(16.1)	193	(16.7)	145	(12.5)			
Model 1 [c,d]	1.00	(Ref)	0.76	(0.61 to 0.95)	0.79	(0.64 to 0.99)	0.55	(0.43 to 0.69)	0.83	(0.73 to 0.92)	**<0.001**
Model 2 [d,e]	1.00	(Ref)	0.80	(0.64 to 1.00)	0.86	(0.68 to 1.08)	0.59	(0.46 to 0.76)	0.86	(0.76 to 0.96)	**0.006**

[a] All values are means (SDs), numbers (%), or relative ORs (95% CI). All estimates were derived from a multivariable logistic regression model. Physical frailty was assessed using the validated Fried phenotype model-based Frailty Screening Index. Bold *p* values are statistically significant ($p < 0.05$).

CI, confidence interval; HSES, high socioeconomic status; LSES, low socioeconomic status; OR, odds ratio; Ref, reference; SD, standard deviation. Q1 through Q4 included the Japanese Food Guide Spinning Top scores of <52.1, 52.1–57.0, 57.1–61.8, and ≥61.9 in women; and <46.6, 46.6–52.2, 52.3–57.5, and ≥57.6 in men; and <50.4, 50.4–56.0, 56.1–61.3, and ≥61.4 in participants with HSES; <48.8, 48.8–54.3, 54.4–59.6, and ≥59.7 in participants with LSES.

[b] Linear trend *p* values were calculated with the likelihood ratio test using continuous variables of adherence scores.

[c] Model 1 was adjusted for age (continuous), sex (female or male), and population density (≥1000 or <1000 people/km^2).

[d] Variables (sex or socioeconomic status) used for subgroup analysis were excluded from the adjustment of covariate variables in the model.

[e] Model 2 was Model 1 with mutual adjustment for body mass index (continuous), physical activity (yes or no), denture use (yes or no), smoking status (never smoker, past smoker, and current smoker), alcohol intake status (every day, sometimes, seldom, or never), educational attainment (<9, 10–12, or ≥13 years), medication use (continuous), living alone (yes or no), socioeconomic status (high or low), green tea consumption (frequency), coffee consumption (frequency), and history of disease (hypertension, diabetes, dyslipidaemia, heart disease, and stroke; yes or no).

Supplementary Table 2. Odds ratios for the Japanese Food Guide Spinning Top adherence score and prevalence of comprehensive frailty defined by the Kihon Checklist, calculated using sex and socioeconomic status stratified multivariate logistic regression [a]

	Quartile of the Japanese food guide Spinning Top score								10 points increment		*p* for trend [b]
	Q1		Q2		Q3		Q4				
Women, n	928		927		927		927				
Mean (SD) score	46.5	(6.2)	54.7	(1.4)	59.5	(1.4)	65.5	(2.8)			
Case [*n* (%)]	464	(50.0)	358	(38.6)	319	(34.4)	264	(28.5)			
Model 1 [c,d]	1.00	(Ref)	0.63	(0.52 to 0.77)	0.53	(0.43 to 0.65)	0.38	(0.31 to 0.47)	0.59	(0.50 to 0.68)	**<0.001**
Model 2 [d,e]	1.00	(Ref)	0.68	(0.55 to 0.85)	0.62	(0.50 to 0.77)	0.47	(0.38 to 0.59)	0.68	(0.59 to 0.78)	**<0.001**
Men, n	829		828		828		828				
Mean (SD) score	40.9	(5.5)	49.6	(1.6)	54.9	(1.5)	61.8	(3.2)			
Case [*n* (%)]	325	(39.2)	271	(32.7)	275	(33.2)	238	(28.7)			
Model 1 [c,d]	1.00	(Ref)	0.78	(0.63 to 0.96)	0.80	(0.65 to 0.98)	0.62	(0.50 to 0.77)	0.81	(0.73 to 0.90)	**<0.001**
Model 2 [d,e]	1.00	(Ref)	0.86	(0.69 to 1.08)	0.93	(0.74 to 1.17)	0.74	(0.59 to 0.94)	0.90	(0.81 to 0.99)	**0.044**
HSES, n	599		599		598		598				
Mean (SD) score	44.3	(6.0)	53.5	(1.6)	58.7	(1.5)	64.8	(2.8)			
Case [*n* (%)]	192	(32.1)	162	(27.1)	151	(25.3)	138	(23.1)			
Model 1 [c,d]	1.00	(Ref)	0.80	(0.60 to 1.06)	0.70	(0.53 to 0.94)	0.57	(0.42 to 0.76)	0.73	(0.61 to 0.86)	**<0.001**
Model 2 [d,e]	1.00	(Ref)	0.85	(0.64 to 1.14)	0.76	(0.56 to 1.03)	0.63	(0.46 to 0.86)	0.77	(0.64 to 0.89)	**<0.001**
LSES, n	1,157		1,157		1,157		1,157				
Mean (SD) score	42.7	(5.9)	51.8	(1.5)	56.9	(1.5)	63.7	(3.2)			
Case [*n* (%)]	538	(46.5)	495	(42.8)	449	(38.8)	389	(33.6)			
Model 1 [c,d]	1.00	(Ref)	0.84	(0.71 to 1.00)	0.69	(0.57 to 0.82)	0.51	(0.43 to 0.62)	0.73	(0.66 to 0.81)	**<0.001**
Model 2 [d,e]	1.00	(Ref)	0.89	(0.74 to 1.07)	0.76	(0.63 to 0.92)	0.58	(0.48 to 0.71)	0.79	(0.71 to 0.87)	**<0.001**

[a] All values are means (SDs), numbers (%), or relative ORs (95% CI). All estimates were derived from a multivariable logistic regression model. Comprehensive frailty was assessed using the validated the Kihon Checklist. Bold *p* values are statistically significant ($p < 0.05$).

CI, confidence interval; HSES, high socioeconomic status; LSES, low socioeconomic status; OR, odds ratio; Ref, reference; SD, standard deviation. Q1 through Q4 include of the Japanese Food Guide Spinning Top scores of <52.1, 52.1–57.0, 57.1–61.8, and ≥61.9 in women; and <46.6, 46.6–52.2, 52.3–57.5, and ≥57.6 in men; and <50.4, 50.4–56.0, 56.1–61.3, and ≥61.4 in participants with HSES; <48.8, 48.8–54.3, 54.4–59.6, and ≥59.7 in participants with LSES.

[b] Linear trend *p* values were calculated with the likelihood ratio test using continuous variables of adherence scores.

[c] Model 1 was adjusted for age (continuous), sex (female or male), and population density (≥1000 or <1000 people/km^2).

[d] Variables (sex or socioeconomic status) used for subgroup analysis were excluded from the adjustment in covariate variables in the model.

[e] Model 2 was Model 1 with mutual adjustment for body mass index (continuous), physical activity (yes or no), denture use (yes or no), smoking status (never smoker, past smoker, and current smoker), alcohol intake status (every day, sometimes, seldom, or never), educational attainment (<9, 10–12, or ≥13 years), medication use (continuous), living alone (yes or no), socioeconomic status (high or low), green tea consumption (frequency), coffee consumption (frequency), and history of disease (hypertension, diabetes, dyslipidaemia, heart disease, and stroke; yes or no).

Supplementary Table 3. Odds ratios for the Japanese Food Guide Spinning Top adherence score and prevalence of the Fried phenotype model subdomains, calculated using multivariate logistic regression [a]

	Quartile of the Japanese food guide Spinning Top score								10 points increment		*p* for trend [b]
	Q1 (*n* = 1756)		Q2 (*n* = 1756)		Q3 (*n* = 1755)		Q4 (*n* = 1755)				
Mean (SD) score	43.2	(5.9)	52.3	(51.6)	57.5	(1.5)	64.1	(3.0)			
**Weight loss**											
Case [*n* (%)]	270	(15.4)	248	(14.1)	226	(12.9)	218	(12.4)			
Model 1 [c]	1.00	(Ref)	0.94	(0.77 to 1.13)	0.85	(0.70 to 1.04)	0.82	(0.67 to 1.00)	0.97	(0.88 to 1.05)	0.416
Model 2 [d]	1.00	(Ref)	0.98	(0.80 to 1.18)	0.91	(0.75 to 1.12)	0.87	(0.71 to 1.07)	0.99	(0.90 to 1.08)	0.838
**Slow gait speed**											
Case [*n* (%)]	1169	(66.6)	1158	(66.0)	1096	(62.5)	1081	(61.6)			
Model 1 [c]	1.00	(Ref)	0.96	(0.83 to 1.12)	0.80	(0.69 to 0.93)	0.71	(0.61 to 0.82)	0.82	(0.75 to 0.88)	**<0.001**
Model 2 [d]	1.00	(Ref)	1.01	(0.86 to 1.18)	0.86	(0.74 to 1.01)	0.79	(0.67 to 0.92)	0.86	(0.79 to 0.93)	**<0.001**
**Cognition**											
Case [*n* (%)]	179	(10.2)	155	(8.8)	141	(8.0)	116	(6.6)			
Model 1 [c]	1.00	(Ref)	0.90	(0.72 to 1.14)	0.84	(0.66 to 1.07)	0.70	(0.54 to 0.90)	0.82	(0.72 to 0.92)	**<0.001**
Model 2 [d]	1.00	(Ref)	0.92	(0.73 to 1.16)	0.90	(0.70 to 1.14)	0.76	(0.59 to 0.99)	0.85	(0.75 to 0.95)	**0.004**
**Exhaustion**											
Case [*n* (%)]	599	(34.1)	549	(31.3)	544	(31.0)	511	(29.1)			
Model 1 [c]	1.00	(Ref)	0.84	(0.72 to 0.97)	0.80	(0.69 to 0.93)	0.69	(0.59 to 0.80)	0.85	(0.79 to 0.91)	**<0.001**
Model 2 [d]	1.00	(Ref)	0.89	(0.76 to 1.04)	0.89	(0.76 to 1.04)	0.78	(0.67 to 0.92)	0.90	(0.83 to 0.97)	**0.003**
**Low activity**											
Case [*n* (%)]	367	(20.9)	330	(18.8)	331	(18.9)	278	(15.8)			
Model 1 [c]	1.00	(Ref)	0.85	(0.72 to 1.00)	0.84	(0.71 to 0.99)	0.66	(0.55 to 0.79)	0.85	(0.77 to 0.92)	**<0.001**
Model 2 [d,e]	1.00	(Ref)	0.90	(0.75 to 1.06)	0.91	(0.76 to 1.09)	0.72	(0.59 to 0.86)	0.88	(0.80 to 0.95)	**0.002**

[a] All values are means (SDs), numbers (%), or relative ORs (95% CI). All estimates were derived from a multivariable logistic regression model. Bold *p* values are statistically significant ($p < 0.05$). Q1 through Q4 include of the Japanese Food Guide Spinning Top score of <49.5, 49.5–54.8, 54.9–60.1, and ≥60.2 scores.
CI, confidence interval; OR, odds ratio; Ref, reference; SD, standard deviation.
[b] Linear trend *p* values were calculated with the likelihood ratio test using continuous variables of adherence scores.
[c] Model 1 was adjusted for age (continuous), sex (female or male), and population density (≥1000 or <1000 people/km^2).
[d] Model 2 was Model 1 with mutual adjustment for body mass index (continuous), physical activity (yes or no), denture use (yes or no), smoking status (never smoker, past smoker, and current smoker), alcohol intake status (every day, sometimes, seldom, or never), educational attainment (<9, 10–12, or ≥13 years), medication use (continuous), living alone (yes or no), socioeconomic status (high or low), green tea consumption (frequency), coffee consumption (frequency), and history of disease (hypertension, diabetes, dyslipidaemia, heart disease, and stroke; yes or no).
[e] Physical activity was excluded from the adjustment of covariate variables in the model.

Supplementary Table 4. Odds ratios for the Japanese Food Guide Spinning Top adherence score and the prevalence of the Kihon Checklist subdomains, calculated using multivariate logistic regression [a]

	Quartile of the Japanese food guide Spinning Top score								10 points increment		*p* for trend [b]
	Q1 (*n* = 1756)		Q2 (*n* = 1756)		Q3 (*n* = 1755)		Q4 (*n* = 1755)				
Mean (SD) score	43.2	(5.9)	52.3	(51.6)	57.5	(1.5)	64.1	(3.0)			
**IADL disability**											
Case [*n* (%)]	186	(10.6)	173	(9.9)	142	(8.1)	99	(5.6)			
Model 1 [c]	1.00	(Ref)	0.79	(0.68 to 0.91)	0.66	(0.57 to 0.76)	0.49	(0.42 to 0.57)	0.71	(0.62 to 0.81)	**<0.001**
Model 2 [d]	1.00	(Ref)	1.01	(0.80 to 1.29)	0.89	(0.69 to 1.15)	0.59	(0.44 to 0.78)	0.79	(0.69 to 0.90)	**<0.001**
**Physical**											
Case [*n* (%)]	392	(22.3)	411	(23.4)	372	(21.2)	371	(21.1)			
Model 1 [c]	1.00	(Ref)	0.97	(0.81 to 1.15)	0.79	(0.66 to 0.94)	0.69	(0.58 to 0.83)	0.86	(0.79 to 0.94)	**<0.001**
Model 2 [d]	1.00	(Ref)	1.03	(0.87 to 1.23)	0.92	(0.76 to 1.10)	0.86	(0.71 to 1.03)	0.93	(0.86 to 1.01)	0.063
**Nutrition**											
Case [*n* (%)]	36	(2.1)	32	(1.8)	21	(1.2)	37	(2.1)			
Model 1 [c]	1.00	(Ref)	0.85	(0.51 to 1.41)	0.55	(0.31 to 0.98)	0.94	(0.57 to 1.54)	0.93	(0.72 to 1.14)	0.493
Model 2 [d]	1.00	(Ref)	0.89	(0.49 to 1.60)	0.49	(0.25 to 0.96)	1.24	(0.67 to 2.27)	0.94	(0.67 to 1.22)	0.671
**Oral**											
Case [*n* (%)]	462	(26.3)	442	(25.2)	399	(22.7)	350	(19.9)			
Model 1 [c]	1.00	(Ref)	0.97	(0.83 to 1.13)	0.86	(0.73 to 1.01)	0.70	(0.59 to 0.82)	0.88	(0.81 to 0.95)	**<0.001**
Model 2 [d]	1.00	(Ref)	1.01	(0.86 to 1.18)	0.96	(0.81 to 1.13)	0.81	(0.68 to 0.97)	0.95	(0.88 to 1.02)	0.136
**Social**											
Case [*n* (%)]	170	(9.7)	143	(8.1)	128	(7.3)	111	(6.3)			
Model 1 [c]	1.00	(Ref)	0.78	(0.61 to 0.99)	0.67	(0.52 to 0.87)	0.55	(0.42 to 0.71)	0.76	(0.66 to 0.86)	**<0.001**
Model 2 [d]	1.00	(Ref)	0.85	(0.62 to 1.03)	0.79	(0.56 to 0.95)	0.68	(0.46 to 0.81)	0.83	(0.73 to 0.94)	**0.003**
**Cognitive**											
Case [*n* (%)]	736	(42.0)	628	(35.8)	616	(35.1)	522	(29.7)			
Model 1 [c]	1.00	(Ref)	0.81	(0.70 to 0.93)	0.80	(0.69 to 0.92)	0.63	(0.54 to 0.72)	0.83	(0.77 to 0.89)	**<0.001**
Model 2 [d]	1.00	(Ref)	0.85	(0.66 to 1.09)	0.90	(0.61 to 1.02)	0.73	(0.51 to 0.89)	0.89	(0.82 to 0.95)	**<0.001**
**Depression**											
Case [*n* (%)]	608	(34.6)	558	(31.8)	506	(28.8)	465	(26.5)			
Model 1 [c]	1.00	(Ref)	0.83	(0.72 to 0.97)	0.71	(0.61 to 0.82)	0.60	(0.51 to 0.70)	0.80	(0.74 to 0.86)	**<0.001**
Model 2 [d]	1.00	(Ref)	0.93	(0.80 to 1.08)	0.83	(0.71 to 0.97)	0.73	(0.62 to 0.86)	0.87	(0.80 to 0.93)	**<0.001**

[a] All values are means (SDs), numbers (%), or relative ORs (95% CI). All estimates were derived from a multivariable logistic regression model. Bold *p* values are statistically significant ($p < 0.05$). Q1 through Q4 include Japanese Food Guide Spinning Top scores of <49.5, 49.5–54.8, 54.9–60.1, and ≥60.2.
CI, confidence interval; OR, odds ratio; Ref, reference; SD, standard deviation.

[b] Linear trend *p* values were calculated with the likelihood ratio test using continuous variables of adherence scores.
[c] Model 1 was adjusted for age (continuous), sex (female or male), and population density (≥1000 or <1000 people/km^2).
[d] Model 2 was Model 1 with mutual adjustment for body mass index (continuous), physical activity (yes or no), denture use (yes or no), smoking status (never smoker, past smoker, and current smoker), alcohol intake status (every day, sometimes, seldom, or never), educational attainment (<9, 10–12, or ≥13 years), medication use (continuous), living alone (yes or no), socioeconomic status (high or low), green tea consumption (frequency), coffee consumption (frequency), and history of disease (hypertension, diabetes, dyslipidaemia, heart disease, and stroke; yes or no).

Supplementary Table 5. Multivariable adjusted odds ratios and 95% confidence intervals of the prevalence of comprehensive frailty according to the adherence score of each component in the Japanese Food Guide Spinning Top [a]

	Quartile of the Japanese food guide Spinning Top score										
	Q1 (*n* = 1756)		Q2 (*n* = 1756)		Q3 (*n* = 1755)		Q4 (*n* = 1755)		1 points increment		*p* for trend [b]
**Grain dishes**											
Case [*n* (%)]	627	(35.7)	604	(34.4)	625	(35.6)	658	(37.5)			
Model 1 [c]	1.00	(Ref)	0.91	(0.78 to 1.05)	0.91	(0.79 to 1.05)	0.89	(0.77 to 1.03)	0.98	(0.96 to 1.00)	0.077
Model 2 [d]	1.00	(Ref)	0.91	(0.78 to 1.07)	0.90	(0.77 to 1.05)	0.85	(0.73 to 0.99)	0.98	(0.96 to 1.00)	0.119
**Vegetable dishes**											
Case [*n* (%)]	746	(42.5)	630	(35.9)	576	(32.8)	562	(32.0)			
Model 1 [c]	1.00	(Ref)	0.74	(0.64 to 0.86)	0.59	(0.51 to 0.68)	0.50	(0.43 to 0.58)	0.90	(0.88 to 0.92)	**<0.001**
Model 2 [d]	1.00	(Ref)	0.80	(0.69 to 0.94)	0.67	(0.57 to 0.79)	0.63	(0.53 to 0.74)	0.94	(0.91 to 0.96)	**<0.001**
**Fish and meat dishes**											
Case [*n* (%)]	671	(38.2)	640	(36.5)	592	(33.7)	611	(34.8)			
Model 1 [c]	1.00	(Ref)	1.07	(0.93 to 1.24)	1.03	(0.89 to 1.19)	1.10	(0.95 to 1.27)	1.01	(0.99 to 1.03)	0.168
Model 2 [d]	1.00	(Ref)	1.02	(0.87 to 1.19)	0.95	(0.81 to 1.11)	1.05	(0.90 to 1.23)	1.00	(0.99 to 1.02)	0.817
**Milk**											
Case [*n* (%)]	734	(41.8)	657	(37.4)	520	(29.6)	603	(34.4)			
Model 1 [c]	1.00	(Ref)	0.95	(0.82 to 1.09)	0.63	(0.54 to 0.73)	0.75	(0.65 to 0.87)	0.96	(0.95 to 0.97)	**<0.001**
Model 2 [d]	1.00	(Ref)	1.01	(0.86 to 1.17)	0.71	(0.61 to 0.84)	0.87	(0.75 to 1.02)	0.98	(0.96 to 0.99)	**0.006**
**Fruits**											
Case [*n* (%)]	772	(44.0)	653	(37.2)	557	(31.7)	532	(30.3)			
Model 1 [c]	1.00	(Ref)	0.72	(0.63 to 0.83)	0.52	(0.45 to 0.60)	0.45	(0.39 to 0.53)	0.91	(0.89 to 0.93)	**<0.001**
Model 2 [d]	1.00	(Ref)	0.83	(0.71 to 0.97)	0.62	(0.53 to 0.72)	0.59	(0.50 to 0.70)	0.94	(0.92 to 0.96)	**<0.001**
**Total energy**											
Case [*n* (%)]	618	(35.2)	586	(33.4)	714	(40.7)	596	(34.0)			
Model 1 [c]	1.00	(Ref)	1.31	(1.11 to 1.55)	1.64	(1.38 to 1.96)	1.05	(0.87 to 1.26)	1.13	(1.05 to 1.22)	**0.001**
Model 2 [d]	1.00	(Ref)	1.26	(1.16 to 1.51)	1.53	(1.26 to 1.86)	1.14	(0.93 to 1.40)	1.13	(1.04 to 1.23)	**0.004**
**Snacks and alcohol**											
Case [*n* (%)]	688	(39.2)	699	(39.8)	587	(33.5)	540	(30.8)			
Model 1 [c]	1.00	(Ref)	1.03	(0.89 to 1.19)	0.70	(0.60 to 0.81)	0.58	(0.50 to 0.68)	0.92	(0.90 to 0.94)	**<0.001**
Model 2 [d]	1.00	(Ref)	0.98	(0.84 to 1.14)	0.71	(0.61 to 0.84)	0.66	(0.56 to 0.78)	0.97	(0.95 to 0.99)	**0.048**
**White to red meat**											
Case [*n* (%)]	646	(36.8)	575	(32.7)	695	(39.6)	598	(34.1)			
Model 1 [c]	1.00	(Ref)	0.86	(0.74 to 0.99)	1.08	(0.93 to 1.25)	0.78	(0.68 to 0.91)	0.98	(0.96 to 1.00)	0.084
Model 2 [d]	1.00	(Ref)	0.87	(0.75 to 1.02)	1.03	(0.88 to 1.20)	0.85	(0.73 to 0.99)	0.99	(0.97 to 1.01)	0.220

[a] All values are numbers (%), or relative ORs (95% CI). All estimates were derived from a multivariable logistic regression model. Comprehensive frailty was assessed using the Kihon Checklist. Bold *p* values are statistically significant ($p < 0.05$). Q1 through Q4 includes the Japanese Food Guide Spinning Top scores of <49.5, 49.5–54.8, 54.9–60.1, and ≥60.2.

CI, confidence interval; OR, odds ratio; Ref, reference.

[b] Linear trend *p* values were calculated with the likelihood ratio test using continuous variables of adherence scores.

[c] Model 1 was adjusted for age (continuous), sex (female or male), and population density (≥1000 or <1000 people/km^2).

[d] Model 2 was Model 1 with mutual adjustment for body mass index (continuous), physical activity (yes or no), denture use (yes or no), smoking status (never smoker, past smoker, and current smoker), alcohol intake status (every day, sometimes, seldom, or never), educational attainment (<9, 10–12, or ≥13 years), medication use (continuous), living alone (yes or no), socioeconomic status (high or low), green tea consumption (frequency), coffee consumption (frequency), and history of disease (hypertension, diabetes, dyslipidaemia, heart disease, and stroke; yes or no).
